# Excessive Activation of Notch Signaling in Macrophages Promote Kidney Inflammation, Fibrosis, and Necroptosis

**DOI:** 10.3389/fimmu.2022.835879

**Published:** 2022-02-25

**Authors:** Tiankui Ma, Xin Li, Yonghong Zhu, Shufan Yu, Tianyan Liu, Xiaodan Zhang, Dong Chen, Shuyan Du, Tong Chen, Shuo Chen, Yanyan Xu, Qiuling Fan

**Affiliations:** ^1^Department of Nephrology, The First Hospital of China Medical University, Shenyang, China; ^2^Department of Nephrology, The Fourth Affiliated Hospital of China Medical University, Shenyang, China; ^3^Department of Centre Laboratory, The First Hospital of China Medical University, Shenyang, China

**Keywords:** diabetic kidney disease, macrophages, kidney inflammation, renal fibrosis, necroptosis, diabetic nephropathy, Notch, NF-κB

## Abstract

Diabetic nephropathy (DN) is one of the main causes of end-stage renal disease (ESRD). Existing treatments cannot control the progression of diabetic nephropathy very well. In diabetic nephropathy, Many monocytes and macrophages infiltrate kidney tissue. However, the role of these cells in the pathogenesis of diabetic nephropathy has not been fully elucidated. In this study, we analyzed patient kidney biopsy specimens, diabetic nephropathy model animals. Meanwhile, we cocultured cells and found that in diabetic nephropathy, damaged intrinsic renal cells (glomerular mesangial cells and renal tubular epithelial cells) recruited monocytes/macrophages to the area of tissue damage to defend against and clear cell damage. This process often involved the activation of different types of macrophages. Interestingly, the infiltrating macrophages were mainly M1 (CD68+iNOS+) macrophages. In diabetic nephropathy, crosstalk between the Notch pathway and NF-κB signaling in macrophages contributed to the polarization of macrophages. Hyperpolarized macrophages secreted large amounts of inflammatory cytokines and exacerbated the inflammatory response, extracellular matrix secretion, fibrosis, and necroptosis of intrinsic kidney cells. Additionally, macrophage depletion therapy with clodronate liposomes and inhibition of the Notch pathway in macrophages alleviated the pathological changes in kidney cells. This study provides new information regarding diabetic nephropathy-related renal inflammation, the causes of macrophage polarization, and therapeutic targets for diabetic nephropathy.

**Graphical Abstract:**
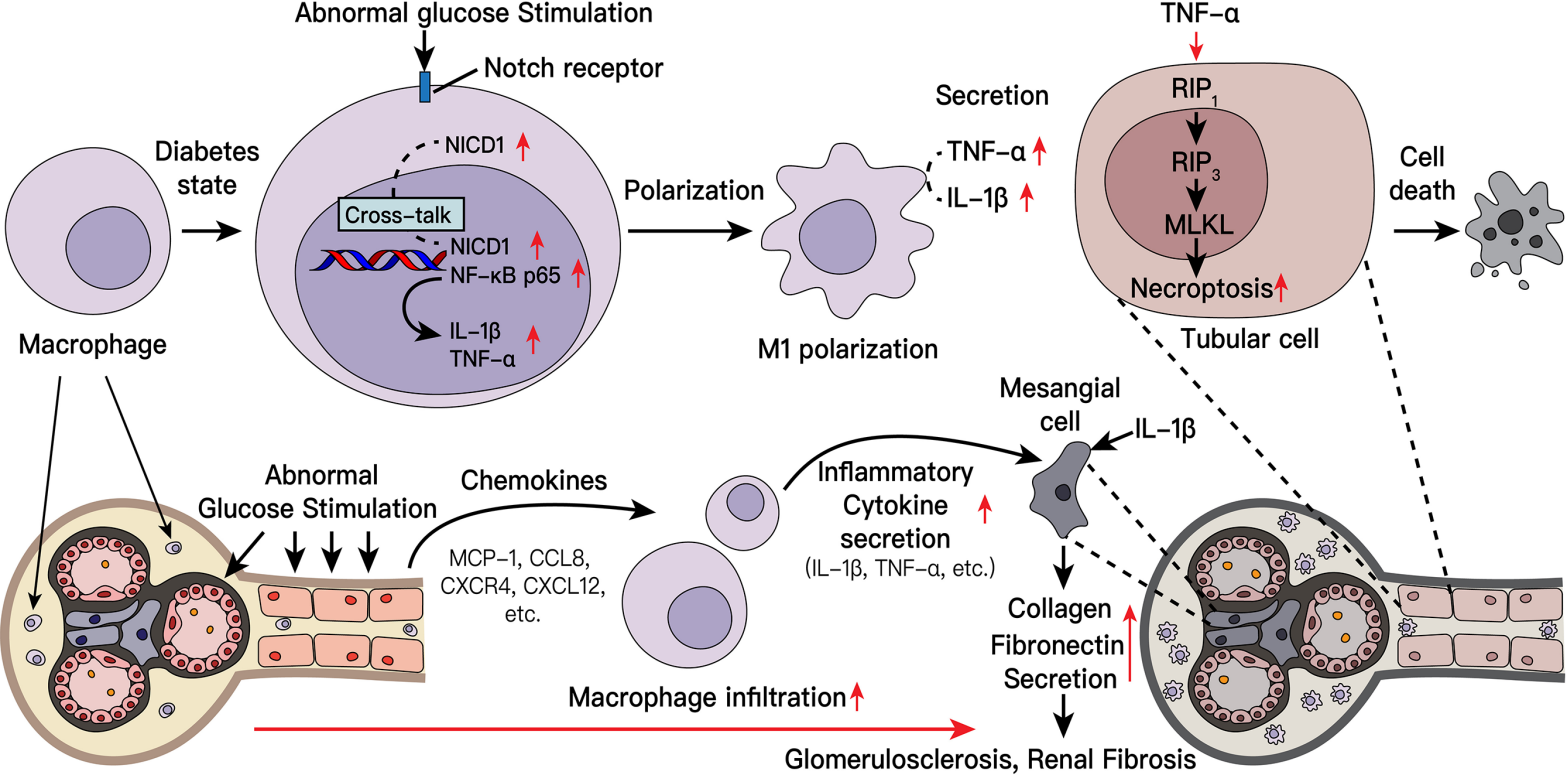


## Introduction

Diabetes has become a global public health problem because of its increasing prevalence. According to the latest statistics from the International Diabetes Federation (IDF), in 2019, approximately 10% of people worldwide had diabetes, with the disease affecting approximately 463 million adults (aged 20-79 years) (1). Diabetes is associated with various microvascular and macrovascular complications, including diabetic nephropathy and diabetic retinopathy etc. (2, 3). According to the United States Renal Data System (USRDS) Annual Data Report, diabetic kidney disease (DKD) is the most common cause of end-stage renal disease (ESRD) (4).

The pathogenesis of diabetic nephropathy is complicated and has not been fully elucidated. According to the traditional view, the pathogenesis of diabetic nephropathy involves genetic factors, hemodynamic effects, serum glucose level and/or lipid metabolism disorders (3, 5). With additional research, the role of macrophages (mφs) in the pathogenesis of diabetic nephropathy has attracted increasing attention. In DKD, numerous monocytes/macrophages accumulate in the glomerulus and renal interstitium. Infiltration by various inflammatory cells and the massive release of inflammatory factors may play an important role in the development of DKD. Several studies have confirmed a large number of infiltrating macrophages in the diabetic kidney, and the number of infiltrating macrophages positively correlates with multiple pathological changes in inherent kidney cells in diabetic nephropathy (6–10).

Macrophages are an important source of inflammatory cytokines (11) and can be classically (M1) or alternatively (M2) activated as needed. Inflammatory M1 macrophages express high levels of proinflammatory cytokines and toxic reactive oxygen intermediates, such as tumor necrosis factor (TNF)-α, interleukin (IL)-1β, IL-6, IL-12, and IL-23, to promote inflammation and/or protect against harmful stimuli and express inducible nitric oxide synthase (iNOS). In contrast, M2 macrophages display immunomodulatory properties, exhibiting various functions, including the production of anti-inflammatory cytokines such as IL-10, IL-4 and IL-13 and the specific expression of arginase-1 (Arg-1) (11–14). Additionally, various proinflammatory factors secreted by polarized macrophages can cause tissue inflammation and aggravate tissue damage (15).

Current research indicates that multiple pathways are involved in the polarization of macrophages (16). Among them, the Notch signaling pathway has been widely reported to be activated in various infection-related macrophages (17–21). The Notch pathway is a highly conserved signaling pathway in various organisms that regulates cell proliferation, metabolism, differentiation, and cell survival (18). In mammals, exist four Notch receptors (Notch1-4) and five ligands (Delta-like ligand [DLL] 1, 3, and 4 and Jagged ligand 1 and 2). Each Notch receptor comprises two functional domains: The Notch extracellular domain (NECD) and the Notch intracellular domain (NICD). The NECD comprises 29-36 epidermal growth factor (EGF) motifs, which mediate the interaction between the ligand and receptor. The NICD has transcriptional activity, it can enter the nucleus and activate downstream pathways (18, 22). In diabetic nephropathy, whether the activation of macrophages is related to the Notch signaling pathway and how the downstream pathway exerts its effect are unclear.

Necroptosis is a newly discovered programmed cell death (PCD) pathway. Necroptosis is driven by a signaling cascade involving receptor interacting protein kinase 1 (RIP1), receptor interacting protein kinase 3 (RIP3), and pseudokinase mixed lineage kinase domain-like protein (MLKL). Following organelle and cellular swelling, dying cells rupture and release their intracellular components (23). Usually, necroptotic cells exhibit the same morphological characteristics as necrotic cells (24). Previous studies have shown that cells undergo necroptosis to fight infection (25). Additional studies have shown that necroptosis plays an important pathogenic mechanism in various diseases, such as myocardial infarction and stroke, atherosclerosis, ischemia-reperfusion injury, pancreatitis, and inflammatory bowel disease (26–28). To date, many studies have reported that the inflammatory factor TNF-α, a physiologically and pathologically significant cytokine, induces necroptosis in tissue cells (26–28). Recent studies have confirmed that necroptosis occurs in several types of kidney diseases, like crystal nephropathy (29), acute renal injury (30) and in podocytes of diabetic nephropathy (31), but the mechanism of necroptosis driven by macrophages in other kidney cells under diabetic nephropathy remains unclear and need to be further verified.

In this study, we analyzed kidney biopsy tissues from patients with diabetic nephropathy, diabetic nephropathy model animals, and cultured cells to explore whether an interaction exists between the Notch pathway and inflammatory NF-κB pathway in macrophages in diabetic nephropathy. Further clarify the relationship between macrophages and kidney intrinsic cell damage in diabetic nephropathy involves the inflammatory response, the increase of extracellular matrix protein, and intrinsic cell death.

## Materials and Methods

### Blood and Kidney Sample Collection From Patients With Diabetic Nephropathy

Between July 2019 and May 2020, patients aged 18-75 years who had undergone renal biopsy at the First Affiliated Hospital of China Medical University were recruited. Biochemical analysis data for the patients were obtained from hospital admission records. In total, 19 patients (10 male and 9 female) were diagnosed with DKD by kidney biopsy. The exclusion criteria were as follows: age <18 years; the presence of other types of kidney disease; pregnancy; infection; genetic disease. The experimental design was approved by the Ethics Committee of the First Affiliated Hospital of China Medical University (approval number: 20202562). Each enrolled patient agreed to participate in the experiment and signed the consent form.

### Animal Experiments

The animal protocol used in this study was approved by the Institutional Animal Care and Use Committee (IACUC) of China Medical University (approval number: 16052M). BKS.Cg-^lepr^db/^lepr^db mice and BKS.Cg-^lepr^db/+ (SPF-grade) mice were purchased from the Institute of Model Animals of Nanjing University and raised in the Laboratory Animal Centre of China Medical University. The mice were allowed to eat and drink freely and were housed under a 12-hour light/dark cycle. When the mice were 8 weeks of age, tail vein blood was collected, and the fasting blood glucose levels were measured to confirm spontaneous hyperglycemia. When the blood glucose is greater than 16.7mM, it is considered to have diabetes. Urine samples were collected when the mice were 10th week of age, and the urine albumin-creatinine ratio (UACR) was measured. If UACR was greater than 3 mg/mmol, it was considered to have diabetic nephropathy. From the 10th week, the macrophage-depletion group was administrated intraperitoneal injection of the macrophage scavenger clodronate liposomes (CL, F70101C, FormuMax, USA) once a week to deplete macrophages. The mice were fasted for more than 8 hours for measuring blood glucose and urine (during which they were allowed to drink water). The body weight was measured weekly, and the tail vein blood glucose levels were measured every four weeks. Additionally, every four weeks, metabolic cages were used to obtain mouse urine samples to test urine creatinine and urine albumin levels. When the mice were 20 weeks of age, they were sacrificed, and blood and tissue samples were collected.

### Kidney Pathology

Human kidney tissues were fixed in formaldehyde-acetic acid-ethanol solution (FAA), and the mouse tissue was fixed in 4% paraformaldehyde. After routine dehydration and embedding, 3-μm sections were obtained and subjected to hematoxylin-eosin (H-E), Masson’s trichrome, periodic acid-Schiff staining (PAS), periodic acid-silver methenamine (PASM), and Congo red staining. Staining was performed according to the manufacturer’s instructions at the Institute of Renal Pathology, The First Affiliated Hospital of China Medical University. A Leica microscope was used to acquire images for subsequent analysis. Glomerulosclerosis index (GSI) was graded on a scale of 0 to 4 (0: normal; 1: involvement of <25% of the glomerulus, 2: involvement of 25–50% of the glomerulus; 3: involvement of 51–75% of the glomerulus and 4: involvement of >75% of the glomerulus). The GSI was obtained by 3 experienced renal pathologists independently scoring 30-50 glomeruli, and the average value was used as the final data (32).

### Cell Culture

The mouse macrophage cell line RAW 264.7, the mouse mesangial cell line SV40 MES-13, and the mouse renal tubular epithelial cell line TCMK-1 were purchased from American Type Culture Collection (ATCC) and cultured in Dulbecco’s modified Eagle’s medium (DMEM; HyClone, USA) containing 10% endotoxin-free fetal bovine serum (FBS; Biological Industries), which had undergone a complement removal process. In this study, we used 5.5 mM glucose culture medium for the normal glucose (NG) condition or 35 mM glucose for the high glucose (HG) condition. Before high glucose stimulation, we synchronized the cells with serum-free medium for 12 hours, then adjusted the glucose concentration to a high glucose level (35mM) and stimulate the cells for 48 hours. The glucose concentration was chosen based on previous studies from our group (33, 34). All cells were cultured at 37°C and 5% CO_2_ for subsequent experiments.

### Cell Transfection

We constructed various Notch1 knockdown siRNAs and transfected them into recipient cells using the jetPRIME^®^ transfection system (Polyplus, USA) according to the manufacturer’s instructions. We used RT-qPCR to identify the siRNA with the highest knockdown efficiency for subsequent studies (**Supplement Figure 5**).

To overexpress NICD1, we obtained the mouse NICD1 protein sequence from previous study (35) and constructed an NICD1 overexpression pcDNA3.1(+) plasmid. After transfection, NICD1 overexpression was verified by WB analysis and RT-qPCR, and the overexpression plasmid was used in subsequent experiments.

### Luciferase Assay

RAW264.7 cells from each group were transfected with an NF‐κB luciferase reporter (D2206; Beyotime, China, **Supplement Figure 4**) using the jetPRIME^®^ transfection system (Polyplus, USA) according to the manufacturer’s instructions. Next, the cells were lysed, and luciferase activity was measured using the Luciferase Reporter Assay System (RG005, Beyotime, China).

### Immunohistochemistry (IHC), Immunofluorescence (IF), and Laser Scanning Confocal Microscope Analysis (LSCM)

For IHC, tissue sections were subjected to routine antigen retrieval, incubated with 3% hydrogen peroxide to block endogenous peroxidase activity, blocked with BSA, incubated with the primary antibody overnight (**Supplement Table 2**, the list of antibodies used in this study and dilution ratio), rinsed with PBS 3 times, incubated with the corresponding secondary antibody, and treated with DAB for color development. After nuclei were counterstained, the sections were sealed with neutral balsam, and images were obtained using a Leica microscope. The staining sections were then reviewed and scored as follows by 2 pathologists: the staining color was scored as no positive staining (negative, 0), light-yellow particle (+, 1), brown-yellow particle (++, 2), and brown particle (+++, 3). The positive staining number score: positive cells with <25% staining was scored as negative staining 1; cells with 25-50% staining was scored as 2; cells with 51-75% staining was scored as 3; and cells with 76-100% staining was scored as 4. The final score was defined as staining color score multiplied by staining number score.

For IF and LSCM, tissue sections were subjected to antigen retrieval, blocked with 3% hydrogen peroxide at room temperature, blocked with BSA, and incubated with the primary antibody overnight at 4°C (**Supplement Table 2**, the list of antibodies used in this study and dilution ratio). After washing 3 times with PBS, the sections were incubated with the corresponding secondary antibody, washed 3 times with PBS, and treated with CY3-Tyramine Signal Amplification (TSA) and/or FITC-TSA. The cell nuclei were counterstained with DAPI, the sections were sealed by using the anti-fluorescence quencher, and images were taken under a Leica fluorescence microscope (for IF) or Nikon Ti-E A1 microscope (for LSCM).

### Terminal Deoxynucleotidyl Transferase-Mediated dUTP Nick-End Labelling (TUNEL) Staining

Kidney sections were subjected to routine deparaffinization, antigen repair with proteinase K, and permeabilization with 0.1% Triton, and then TDT enzyme, dUTP, and buffer mixture were added to the sections according to the instructions of the TUNEL kit (C1086; Beyotime, China). After incubation for 2 hours in a 37°C incubator, DAPI was used to counterstain the nuclei. The sections were sealed with anti-fluorescence quencher, and images were captured under a Leica fluorescence microscope.

### Flow Cytometry

In total, 1×10^5^ resuspended cells were collected according to the manufacturer’s instructions, and Annexin V-FITC and PI staining solution were added for staining. After incubation at room temperature for 20 minutes in the dark, a BD FACSVia flow cytometry system was used for detection. The test results were analyzed using FlowJo 10 software.

### Transmission Electron Microscopy (TEM)

Pieces of mouse kidney tissues (1 mm^3^) were rinsed 3 times in PBS, immediately placed in Glutaraldehyde Fixed Solution (2.5%, electron microscopy grade) 4°C overnight, then rinsed 3 times in PBS, placed in 1% osmium acid and fixed at room temperature in dark for 2 hours. After fixation, the tissues were dehydrated in gradient alcohol solutions. The dehydrated samples were embedded in embedding agent and polymerized for 48 hours. The resin blocks were cut to ultrathin sections. Then the sections were stained and observed under a transmission electron microscope (Hitachi HT7800, Japan), and images were collected for analysis.

### RNA Extraction and Real-Time Quantitative Polymerase Chain Reaction (RT-qPCR)

Total RNA was extracted from animal tissues and cells using a Qiagen RNeasy Kit (74104, Qiagen, Germany) according to the manufacturer’s instructions. The RNA was reverse transcribed using the Takara PrimeScript™ RT reagent Kit with gDNA Eraser (RR047; Takara Co., Japan), we constructed specific primers (**Supplement Table 1**, the primer sequences), and real-time quantitative PCR was performed using Takara TB Green^®^ Premix Ex Taq™ II (RR820, Takara Co., Japan). The 2-delta delta CT method was used to calculate the relative expression levels, and each group of experiments was repeated more than 6 times.

### Western Blot (WB) and Co-Immunoprecipitation

Total protein was extracted using RIPA lysis buffer. For co-immunoprecipitation, the lysate containing 200 μg of total protein and specific antibody (1 μg) were incubated overnight at 4°C with continuous rotation. After that, add 50 μl protein A+G agarose beads (P2055, Beyotime co. China) and incubate for 3 hours at 4°C. The beads were washed five times with lysis buffer. Resuspend the pelleted beads in 30 μl 2X SDS sample loading buffer and boil at 95°C for 10 minutes. The proteins were then separated using SDS-PAGE. Immunoblotting was performed using specific antibodies (**Supplement Table 2**, the list of antibodies used in this study and dilution ratio). Semiquantitative analysis was conducted using ImageJ software. All the results were collected from experiments that were repeated more than 6 times.

### Statistical Analysis

Data with a normal distribution are presented as the mean ± standard deviation. Data from multiple groups were compared using one-way ANOVA, and differences between groups were subjected to Fisher’s least significant difference test for multiple comparisons. Differences were considered significant at p<0.05. SPSS 23.0 statistical software was used for analyses.

## Results

### The Kidneys of Patients With Diabetic Nephropathy Exhibit Macrophage Cell Infiltration and Pathological Damage

**Figure 1:**
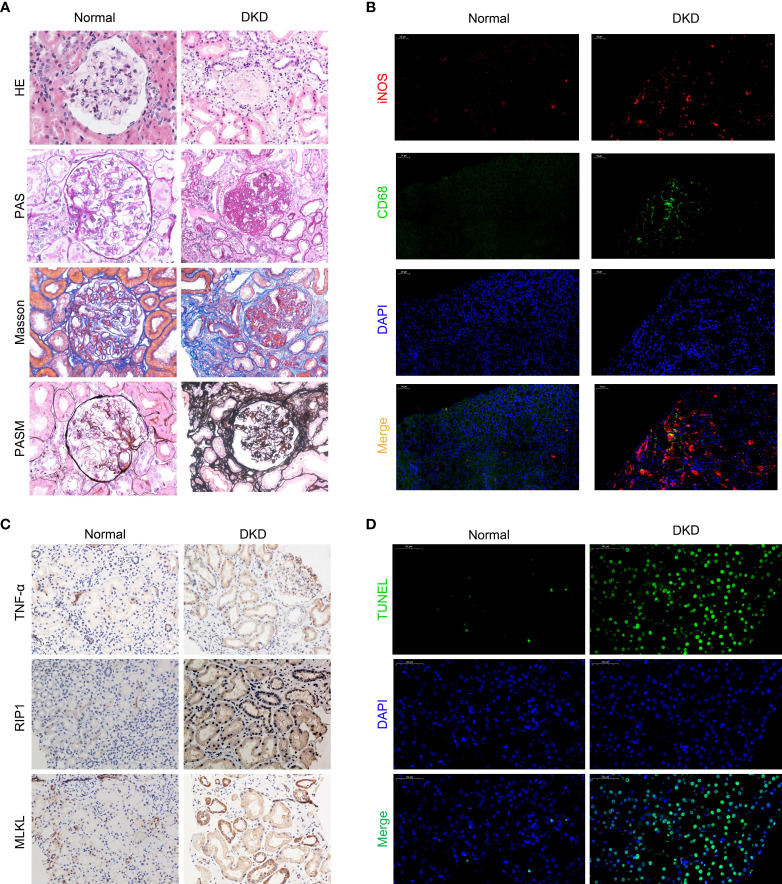
Macrophages infiltrate the kidneys of patients with diabetic kidney disease and are closely related to cell death. **(A)** Staining of kidney specimens included hematoxylin-eosin staining (H-E), periodic acid-Schiff staining (PAS), Masson’s trichrome staining (Masson), and periodic acid-silver methenamine staining (PASM). Magnification: ×400 **(B)** CD68/iNOS double immunofluorescence staining. Magnification: ×200 **(C)** Immunohistochemistry of kidney samples. Magnification: ×400. **(D)** TUNEL method to detect cell death in the kidney. Magnification: ×400.Normal, normal control; DKD, diabetic kidney disease.

**Figure 2:**
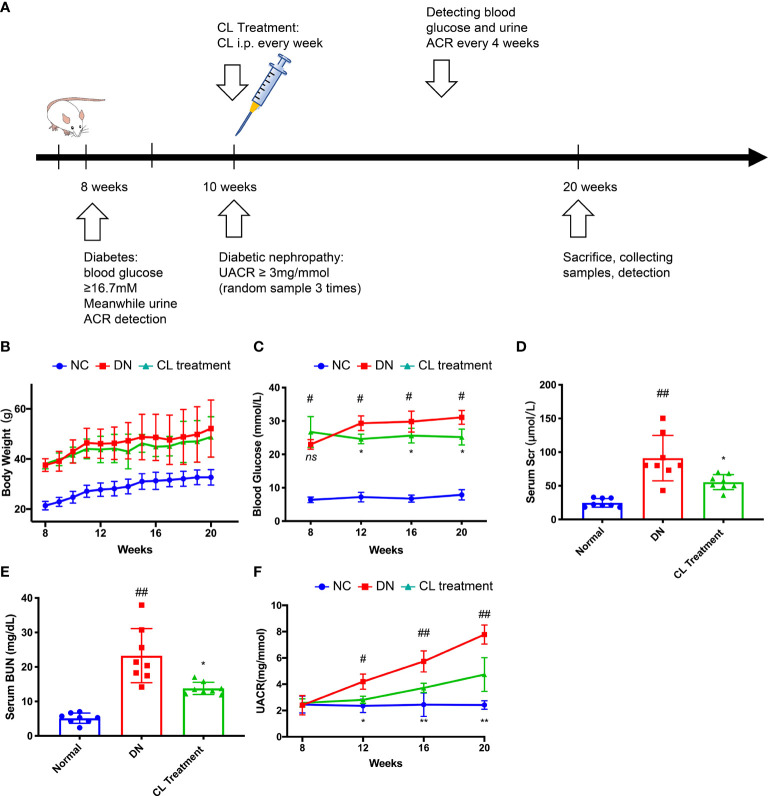
Macrophage depletion alleviates blood glucose, improves kidney function and relieves albuminuria in diabetic mice. **(A)** Schematic of the mouse experimental protocol. **(B)** Body weight of the mice. **(C)** Blood glucose levels of the mice. **(D)** Serum creatinine levels of the mice. **(E)** Blood urea nitrogen (BUN) levels of the mice. **(F)** Urine albumin-creatinine ratios of the mice. NC, normal control group (db/m); DN, DN group (db/db); CL, CL treatment (db/db+CL treatment). ^#^p < 0.05 *vs.* the normal group (db/m), ^##^p < 0.01 vs. the normal group (db/m), *p < 0.05 vs. the DN group (db/db), **p < 0.01 vs. the DN group (db/db) ns, no significance.

We recruited patients with diabetic nephropathy that was confirmed by kidney pathological biopsy from July 2019 to May 2020 at The First Hospital of China Medical University. The patients, 10 of whom were male and 9 of whom were female, were aged 48.9 ± 18.3 years. The average serum creatinine level was 328.7 ± 198.2 µmol/L, and the average estimated glomerular filtration rate (eGFR) was 15.2 ± 14.4 ml/min/1.73 m^2^. We evaluated kidney biopsy specimens from the patients and used kidney tissues from patients undergoing nephrectomy due to trauma as normal controls. We observed infiltrating monocytes/macrophages in the kidney tissues of patients with diabetic kidney disease (**Figure 1A** H-E and **Figure 2B**). Analysis of mesangial cells and the mesangial matrix revealed diffuse hyperplasia, mainly of the matrix (H-E and PAS staining), and Masson’s staining showed significant interstitial fibrosis (**Figure 1A**). We also found granular degeneration and vacuolar degeneration of renal tubules, shedding of the brush border of renal tubular epithelial cells, atrophy of tubular cells and tubular cell death (**Figure 1A**).

### Inflammatory M1 Macrophages Infiltrate the Kidneys of Patients With Diabetic Nephropathy

To investigate the polarized phenotype of macrophages, paraffin sections of tissues from patients with DKD were assessed by double immunofluorescence (IF) staining. Many infiltrating M1 macrophages (CD68+/iNOS+) were found in the interstitium of the patients’ renal tissues (**Figure 1B**). The normal control tissues showed little or no CD68 and/or iNOS costaining (**Figure 1B**). Additionally, fewer M2 macrophages (CD68+/Arg-1+) were found in the tissues from patients with diabetic nephropathy (**Supplement Figure 1**). All together, these results indicate that many macrophages infiltrate the kidney tissues of patients in DKD and the infiltrating macrophages are mainly M1 macrophages.

### Necroptosis Accompanies Renal Tubular Cell Death in Patients With Diabetic Kidney Disease

Pathological examination of the kidney revealed that some patients with DKD exhibited renal tubule degeneration and cell death. To determine the underlying cause, we performed TUNEL staining and used IHC to assess the activity of the RIP1/MLKL necroptosis pathway in kidney tissue (**Figures 1C, D**). These results confirmed that renal tubular cells underwent necroptosis during DKD.

### Macrophage Depletion Improves Urine Protein Levels and the Renal Function of db/db Mice

To verify our clinical findings, we used classic type 2 diabetes model animals, BKS.Cg-^lepr^db/^lepr^db mice (db/db n=10) for follow-up studies (**Figure 2A**). The blood glucose levels of the db/db mice were significantly higher than those of the normal control mice (BKS.Cg-^lepr^db/+, db/m, n=10) at the 8 weeks, and proteinuria was observed at 10 weeks. Proteinuria was accompanied by a significant increase in the UACR, which was higher in the db/db mice than that in the normal control mice (db/m) (**Figure 2F**). Furthermore, we constructed an animal model by chronically depleting macrophages in db/db mice. Specifically, we administered CL weekly by intraperitoneal injection beginning at the age of the 10 weeks to chronically deplete macrophages from tissues (**Figure 2A**). Interestingly, after depleting macrophages, the blood glucose and proteinuria levels of the mice were significantly improved compared with the db/db group (**Figures 2C, F**). Then, we collected serum samples from mice at the 20 weeks and found that the serum creatinine and urea nitrogen levels of the mice were also improved compared with the diabetic nephropathy (db/db) group (**Figures 2D, E**). However, the body weights of the mice did not change, which is similar to that of a previous study (**Figure 2B**) (36).

**Figure 3:**
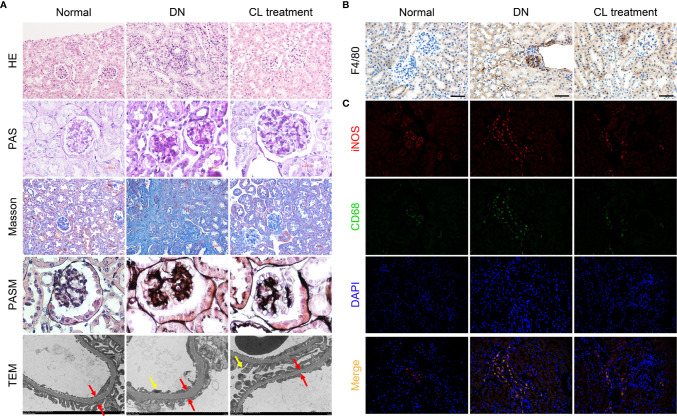
Macrophage depletion improves kidney damages and reduces M1 macrophage infiltration in diabetic mice. **(A)** Histopathologic Staining and transmission electron microscopy (TEM) of mouse kidney tissue. Hematoxylin-eosin (H-E) staining (magnification: 200×); periodic acid-Schiff (PAS) staining (magnification: 400×); Masson’s trichrome (Masson) staining (magnification: 200×); periodic acid-silver methenamine (PASM) staining (magnification: 400×); transmission electron microscopy images (magnification: 4000×). **(B)** F4/80 immunohistochemical staining of the mouse kidney. Original magnification: 200×. **(C)** CD68/iNOS coimmunofluorescence staining of mouse kidney tissue. Original magnification: 400×. Normal: db/m group, DN: db/db group, CL Treatment: db/db + clodronate liposome treatment.

Meanwhile, Depletion of macrophages significantly alleviates pathological damage of kidney tissue in mice with diabetic nephropathy. To explore the effect of macrophages on pathological damage in diabetic kidney tissue, we collected the kidney samples of mice from each group, and subjected the paraffin section to H-E, PAS, Masson, and PASM staining for pathological analysis. The results showed mononuclear/macrophage cell infiltration (**Figure 3A**; H-E staining), mesangial cell proliferation, mesangial matrix secretion, and glomerulosclerosis index (GSI) score were significantly increased in the kidneys of diabetic mice (**Figure 3A**; PAS staining & **Supplement Figure 3**). Masson’s staining also showed that the area of the tubular interstitial matrix increased, and the degree of fibrosis increased compared with the normal control group (db/m) (**Figure 3A**; Masson’s staining). Interestingly, macrophage depletion therapy (CL treatment) significantly improved the abovementioned pathological changes. Transmission electron microscopy (TEM) images showed that the basement membrane was thickened (**Figure 3A**; TEM; red arrow), foot process fusion and abolished in the diabetic group (**Figure 3A**; TEM; yellow arrow). CL treatment also alleviated these pathological changes, as mentioned above (**Figure 3A** TEM).

### Macrophages Infiltrated in the Kidneys of Diabetic Nephropathy Mice Are Mainly the M1 Phenotype

To further investigate the polarization of macrophages in the mouse kidney, we subjected mouse kidney paraffin sections to IHC for F4/80 and double IF for CD68/iNOS (M1 macrophage markers) and CD68/Arg-1 (M2 macrophage markers) to detect infiltrating macrophages in mouse kidneys and determine their polarization states. Infiltrating macrophages (F4/80+ and CD68+) were found in the glomeruli and renal tubules area (**Figures 3B, C**) in the diabetic nephropathy group compared with those in the normal control group. IF costaining showed that most of the infiltrating macrophages were M1 macrophages (**Figure 3C**), and M2 macrophages were relatively rare (**Supplement Figure 2**).

### Macrophage Depletion Reduces the Expression Levels of Chemokines in Kidney Tissue

**Figure 4:**
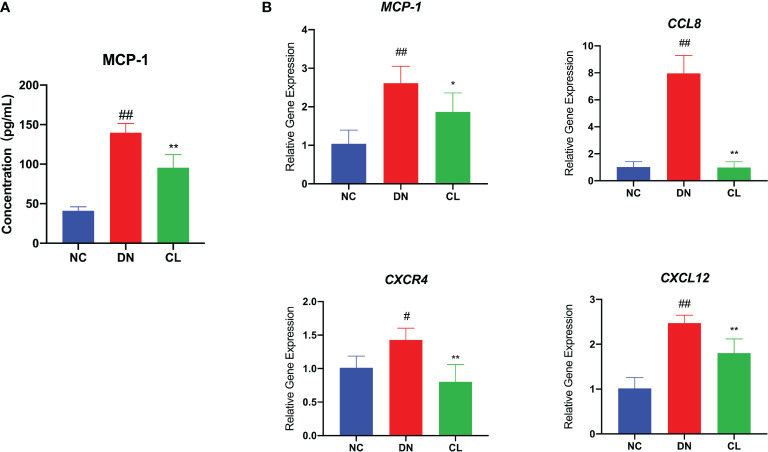
Macrophage depletion significantly reduces the levels of chemokines in blood serum and tissues. **(A)** MCP-1 level in mouse serum. **(B)** mRNA expression of various chemokines in mouse kidney tissue. NC, normal control group (db/m); DN, diabetic nephropathy group (db/db), CL treatment: db/db + clodronate liposome treatment. ^#^p < 0.05 vs. the normal group, ^##^p < 0.01 vs. the normal group, *p < 0.05 vs. the DN group, **p < 0.01 vs. the DN group.

**Figure 5:**
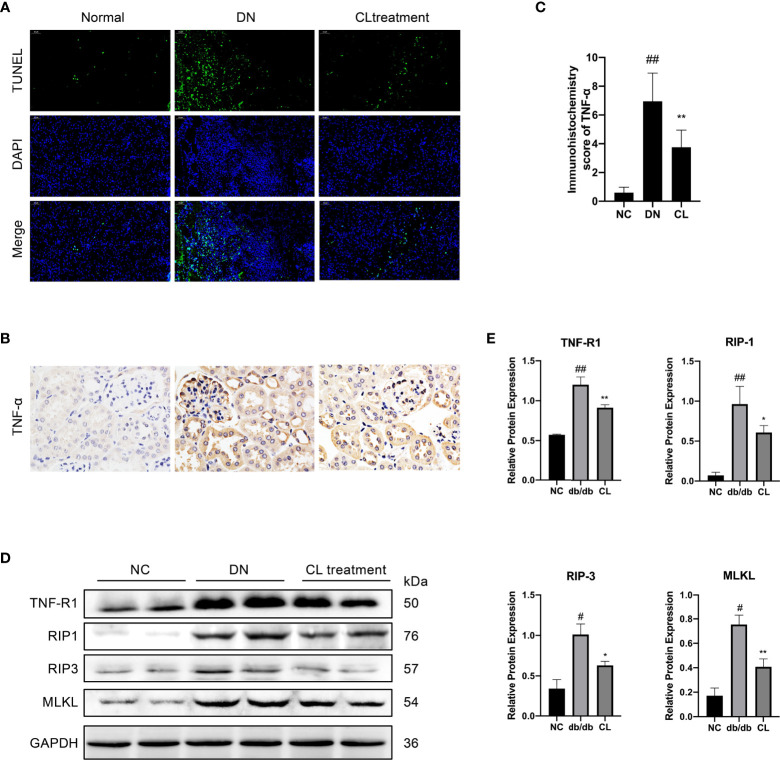
Macrophage depletion reduces the necroptosis of renal tubular cells in diabetic mice. **(A)** TUNEL assay to detect cell death in renal tubulointerstitium of mouse, with counterstaining of cell nuclei (DAPI, blue). Original image magnification: 200×. **(B)** Immunohistochemical staining shows the levels of TNF-α in kidney tissue. Original image magnification: 200×. **(C)** Immunohistochemistry score of TNF-α. **(D, E)** Necroptosis pathway proteins RIP-1, RIP-3, and MLKL in the tissue were detected using western blotting, and GAPDH was used as the loading control. NC: normal control group (db/m), DN: diabetic nephropathy group (db/db), CL treatment: db/db + clodronate liposome treatment. ^#^p < 0.05 vs. the normal group (db/m), ^##^p < 0.05 vs. the normal group (db/m), *p < 0.05 vs. the DN group (db/db), **p < 0.01 vs. the DN group (db/db).

To further explore the causes of macrophage infiltration in kidney tissue, we performed enzyme-linked immunosorbent assay (ELISA) of the chemokine monocyte chemoattractant protein-1 (MCP-1) in mouse blood serum and RT-qPCR analysis of the chemokines monocyte chemoattractant protein-1 (MCP-1), chemokine ligand 8 (CCL8), chemokine (C-X-C motif) receptor 4 (CXCR4), and chemokine (C-X-C motif) ligand 12 (CXCL12) in mouse kidney tissues. The levels of chemokines were significantly increased in the diabetic nephropathy group compared with those in the control group (**Figures 4A, B**). After CL treatment, the levels of chemokines in the kidney decreased significantly (**Figures 5A, B**). This finding suggests that macrophages may be key players in the recruitment of inflammatory cells.

### Macrophage Depletion Alleviates Renal Tubular Necroptosis

Through pathological staining of tissue, we also found that in diabetic nephropathy, the renal tubular cells of the mice showed vacuolar degeneration, death, and shedding (**Figure 3A**). A significant increase was also found in the number of TUNEL-positive cells (**Figure 5A**). Subsequently, we subjected mouse kidney paraffin sections to IHC and found that the TNF-α level in tubular cells in the diabetic nephropathy group was significantly increased (**Figures 5B, C**). Western blot (WB) analysis also showed that the necroptosis pathway-related molecules TNF-αR, RIP-1, RIP-3, and MLKL were expressed in the diabetic kidney and increased significantly (**Figure 5C**). After injection of CL to deplete macrophages, the levels of TNF-α and necroptosis pathway-related molecules TNF-αR/RIP1/RIP3/MLKL in renal tissues were significantly decreased (**Figures 5D, E**).

### Renal Inflammation and Fibrosis Improved After Macrophage-Depletion Therapy

**Figure 6:**
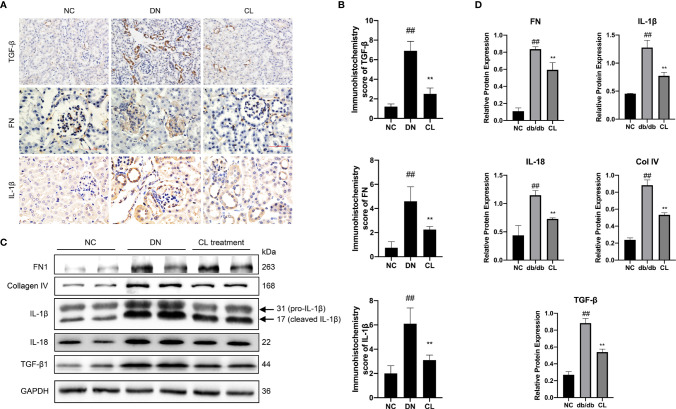
Macrophage depletion reduces the level of inflammation and fibrosis in the kidneys of diabetic mice. **(A, B)** Immunohistochemistry and Immunohistochemistry score were used to evaluate the levels of the inflammation and fibrosis indicators IL-1β, TGF-β, and FN in mouse kidney tissue. **(C, D)** Western blot and semiquantitative analyses of the levels of inflammation and fibrosis indicators in the kidney tissue of mice. NC, normal control group (db/m); DN, diabetic nephropathy group (db/db), CL treatment: db/db + clodronate liposome treatment. ^##^p < 0.01 vs. the normal group (db/m), **p < 0.01 vs. the DN group (db/db).

Immunohistochemical staining of mouse kidney paraffin sections and WB analysis of kidney extracted proteins revealed that the levels of the inflammation-indicating factors IL-1β and IL-18 in diabetic nephropathy mouse kidney tissues increased significantly, and the levels decreased significantly after CL treatment. These results indicate that macrophage-depletion therapy alleviates tissue inflammation (**Figure 6**). Previous research has confirmed that fibrosis is a manifestation of persistent inflammation (37). Next, we examined the levels of transforming growth factor beta (TGF-β), extracellular matrix proteins collagen IV (Col IV) and fibronectin (FN) in the tissues (**Figure 6**). WB and IHC revealed that the levels of Col IV and FN in diabetic nephropathy tissues increased significantly (**Figures 6C, D**). Macrophage depletion significantly improved the levels of Col IV, reduced TGF-β expression and the level of FN in the glomeruli compared with those in the diabetic nephropathy group (**Figure 6**).

### The Notch Pathway Plays an Important Role in Proinflammatory Macrophage Polarization in Diabetic Kidneys

**Figure 7:**
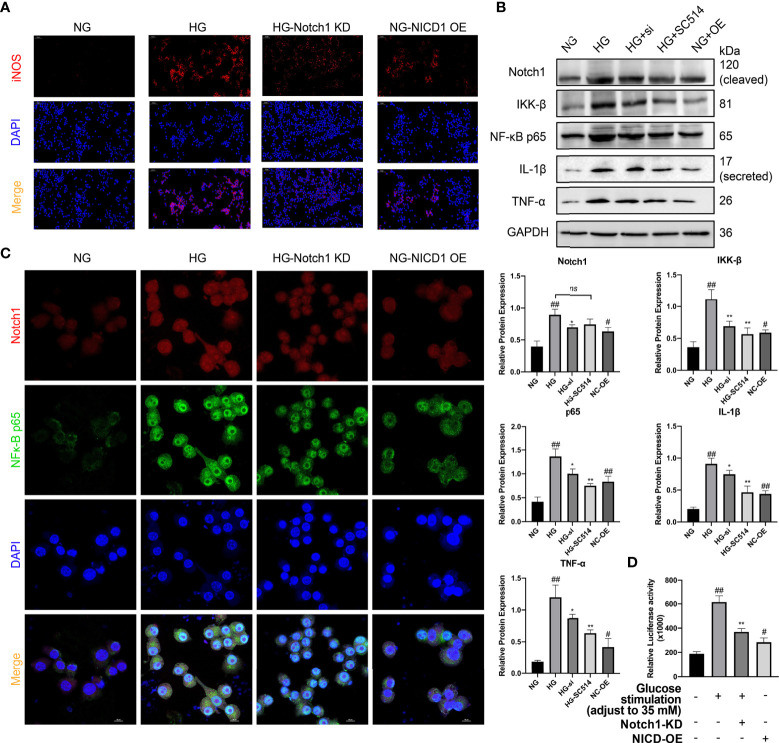
Activation of the Notch signaling pathway plays a crucial role in the M1 polarization of macrophages in HG stimulation. **(A)** Immunofluorescence (IF) of inducible nitric oxide synthase (iNOS). **(B)** Western blot and semiquantitative analyses. **(C)** Confocal microscopy analysis of Notch1 and NF-κB p65 co-IF staining, magnification: 1000×. **(D)** Luciferase Activity of NF-κB-responsive luciferase reporter gene in HG stimulated Raw 264.7 cells. NG, normal glucose; HG, high glucose; Notch KD/si, Notch1 Knockdown; NICD-OE, NICD over expression. ^#^p < 0.05 vs. the normal glucose (NG) group, ^##^p < 0.05 vs. the normal glucose (NG) group. *p < 0.05 vs. the high-glucose (HG) group, **p < 0.01 vs. the high-glucose (HG) group. ns, no significance.

As mentioned, the Notch signaling pathway plays an important role in macrophage polarization and phenotype maintenance. To further explore whether the polarization of renal macrophages under high glucose is related to the Notch pathway, we cultured classic mouse macrophage line RAW264.7 *in vitro*. Under stimulation with HG, macrophages underwent M1 polarization, as indicated by increased expression of iNOS, TNF-α, and IL-1β, and the expression level of Notch1 in macrophages significantly increased compared with those cultured in normal glucose (**Figures 7A, B**).

NICD, an activation product of the Notch signaling pathway, can promote the expression of various nuclear receptors and target genes. Members of the NF-κB family, as a family of nuclear receptors, are likely to be affected by Notch pathway activation. Through WB analysis, we found that the expression of the classical NF-κB molecules IκB kinase β (IKK-B) and NF-κB p65 was significantly upregulated in the cells (**Figure 7B**). Additionally, WB analysis revealed that the expression of the downstream inflammatory factors IL-1β and TNF-α was significantly higher in the cells cultured under HG conditions than in the normal control (**Figure 7B**).

To further explore whether Notch pathway activation influences NF-κB expression in HG stimulated macrophages, we constructed 3 siRNAs to knock down the Notch1 gene and selected one siRNA with the strongest effect one (siRNA #3) for follow-up experiments (**Supplement Figure 5**). Next, we assessed the activity of the NF-κB signaling pathway. After NICD1 activity was inhibited, the expression of the NF-κB pathway-related molecules IKK-B and p65 and the downstream inflammatory factors IL-1β and TNF-α was significantly downregulated. However, the NF-κB/IKK-B inhibitor SC-514 (10 µM; SF0029; Beyotime, China) had little effect on the expression of Notch1. Additionally, we constructed a plasmid to specifically overexpress NICD1. After overexpression, even in NG culture medium, macrophages were tending to the M1-like-phenotype, as in HG medium, and the levels of p65 and IKK-B were increased. Additionally, the levels of the downstream inflammatory factors TNF-α and IL-1β were significantly increased (vs. the normal control group) (**Figure 7B**).

To explore this mechanism underlying this phenomenon induced by exposure to HG conditions, we transfected an NF-κB luciferase reporter gene plasmid (D2206; Beyotime, China; **Supplement Figure 4**) into RAW264.7 cells to assess the mechanism of crosstalk between Notch1 and NF-κB under HG conditions. Using a luciferase reporter system, we found that after Notch1 was knocked down under HG culture conditions, NF-κB activity was significantly reduced, as shown in the **Figure 7D**. However, after NICD was overexpressed, NF-κB pathway activity was significantly enhanced, even under NG conditions (**Figure 7D**). Meanwhile, the interaction between Notch and NF-κB p65 was also detected by reciprocal co-immunoprecipitation(co-IP). The results showed that Anti-Notch1 monoclonal antibody co-precipitated p65, while anti-NF-κB p65 monoclonal antibody also co-precipitated Notch1. More convincingly, Notch1 knockdown decreased whereas NICD overexpression increased the association between Notch1 and NF-κB p65 (**Supplement Figure 6**).

Additionally, we performed double IF staining for Notch1 and NF-κB p65 and used laser scanning confocal microscopy (LSCM) for cytoplasmic and nuclear localization analysis. Notch1 expression increased significantly under HG conditions and was accompanied by a significant increase in phosphorylated p65 that entered the nucleus. After Notch1 was knocked down, the fluorescence intensity of p65 decreased significantly. Furthermore, after NICD was overexpressed in cells cultured in NG, the nuclear p65 level also increased significantly. The above research results indicate that Notch1 overexpression can activate NF-κB p65, a key molecule of the NF-κB pathway, and then mediate the expression of its downstream inflammatory pathway members (**Figure 7C**).

### Mesangial Cells Cocultured With Macrophages Aggravate the Inflammatory Response and Extracellular Matrix Secretion

**Figure 8:**
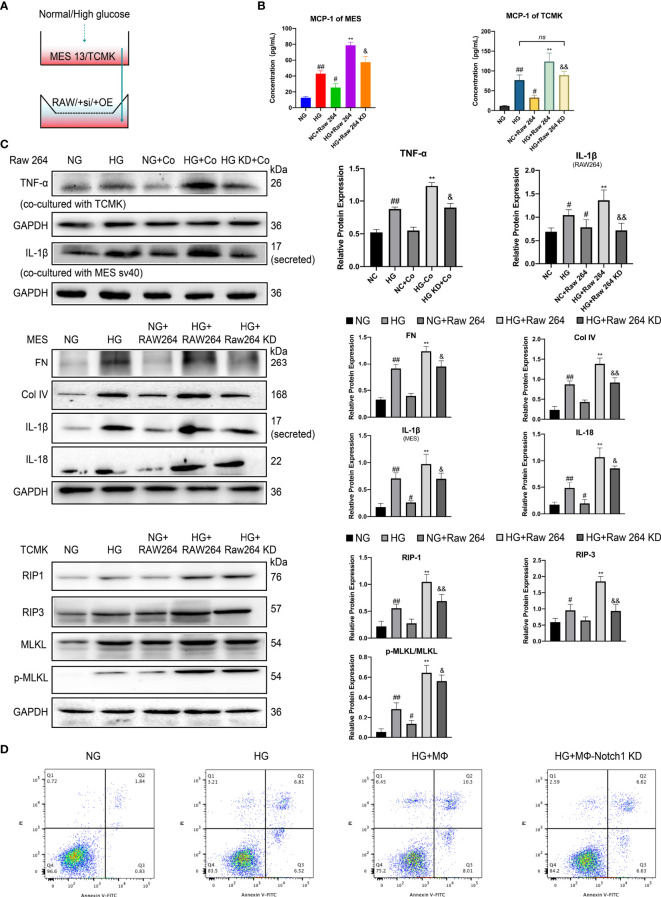
Coculture of macrophages and intrinsic renal cells under high glucose condition significantly aggravates inflammation, fibrosis, and necroptosis of intrinsic renal cells. **(A)** Schematic of the coculture experiments. **(B)** The concentration of MCP-1 in the supernatants of different group cells was determined by ELISA. **(C)** Western blot and semiquantitative analyses. **(D)** Cell death was assessed by Annexin V-FITC/PI staining and analyzed by flow cytometry. NG, normal glucose; HG, high glucose; KD, knockdown; OE, over expression; Co, coculture. ^#^p < 0.05 vs. the normal glucose (NG) group, ^##^p < 0.01 vs. the normal glucose (NG) group, **p < 0.01 vs. the high-glucose (HG) group, ^&^p < 0.05 vs. the high-glucose coculture group, ^&&^p < 0.01 vs. the high-glucose coculture group. ns, no significance.

Subsequently, we explored the mechanism of the interaction between macrophages and renal intrinsic cells under HG conditions *in vitro*. We cocultured RAW264.7 macrophages and intrinsic renal cells (SV40 MES-13 mouse mesangial cells and TCMK-1 mouse renal tubular epithelial cells) in a Transwell^®^ system (Corning, USA) *in vitro* (**Figure 8A**) under normal or HG conditions. We also performed single culture systems of mesangial cells and tubular cells under normal or HG conditions.

In macrophages and mesangial/tubular cells cocultured group under HG conditions, the level of MCP-1 in the cell culture supernatant was increased significantly (vs. that in the single-cultured group) (**Figure 8B**). Additionally, we extracted protein from the RAW264.7 cells of each group for WB analysis and found that the protein expression level of IL-1β was also significantly increased in the coculture group compared with that in the single-culture group (**Figure 8C**). Next, we extracted protein from the cocultured mesangial (MES) cells. In MES cocultured under HG conditions, the levels of the inflammatory factors IL-1β and IL-18 were significantly increased (vs. those in cocultured with macrophages under NG conditions) (**Figure 8C**). Additionally, the expression levels of the extracellular matrix proteins FN and Col IV also increased significantly in mesangial cells (**Figure 8C**). After Notch1 was knocked down in macrophages with siRNA, the levels of the inflammatory factors IL-1beta and IL-18 and the extracellular matrix proteins FN and Col IV in mesangial cells cocultured under HG conditions were significantly decreased (vs. those in cocultured with macrophages under HG) (**Figure 8C**).

### HG Activate Necroptosis Pathway in Renal Tubular Cell *In Vitro*

Next, we conducted *in vitro* studies focus on the death of renal tubular cells, which was found in human kidney pathological biopsy and mouse kidney tissue sections as described before. To investigate the mechanism of TCMK-1 cells death induced by HG, we examined the activation of RIP1/RIP3/MLKL necroptosis pathway. The results showed that after treatment with HG, the level of necroptosis markers RIP1, RIP3, MLKL and phospho-MLKL (p-MLKL, phospho S345) was increased significantly (vs NG) (**Supplement Figure 7**).

To further confirm the mechanism of renal tubular necroptosis in response to HG stimulation, we analyzed the necroptosis pathway with using the pan-caspase inhibitor benzyloxy carbonyl-Val-Ala-Asp-fluoromethyl ketone (z-VAD-fmk, C1202, Beyotime, China) and necroptotic inhibitor Necrostatin-1 (Nec-1, SC4359, Beyotime, China). Western blots showed necroptosis markers RIP1, RIP3, MLKL and p-MLKL expressions were significantly elevated after co-treatment with HG + 20 μ M z-VAD-fmk group compared to the cell treated only with HG (**Supplement Figure 7**). On the contrary, the treatment of cells with 50 μ M Nec-1 significantly inhibit the expression of RIP1, RIP3, MLKL and p-MLKL, but not effect caspase3. Interestingly, z-VAD-fmk induced cell necroptosis can be rescued by Nec-1(**Supplement Figure 7**). These results suggested that the inhibition of apoptosis by z-VAD-fmk could promote the necroptosis of cells induced by HG.

### Macrophages Aggravate the Necroptosis of Renal Tubular Cells in HG

Furthermore, We cocultured RAW264.7 and TCMK-1 under HG conditions (**Figure 8A**). TNF-α is a widely recognized cytokine that mediates cellular necroptosis (38, 39). Then, we extracted protein from RAW264.7 cells from each group and used WB analysis to determine the TNF-α level in each group. Macrophages produced a large amount of TNF-α under HG stimulation (vs. NG), and the TNF-α level in the macrophages cocultured with renal tubular cells under HG conditions was higher than that in macrophages cocultured with tubular cells under NG (**Figure 8C** RAW264). Additionally, after Notch1 was knocked down in macrophages, the level of TNF-α decreased significantly (vs. macrophages under HG conditions). Furthermore, we cocultured Notch1 knockdown macrophages with tubular cells, and the level of TNF-α was significantly reduced (vs. macrophages cocultured with TCMK-1 under HG) (**Figure 8C** RAW264).

To exclude the involvement of apoptosis in renal tubular cell death and more clearly assess necroptosis of renal tubular cells, we pretreated TCMK-1 mouse renal tubular epithelial cells for 2 hours with the z-VAD-fmk (20 µM). Subsequently, the cells were routinely cultured or cocultured with macrophages. WB detected the levels of classic necroptosis pathway molecules RIP1/RIP3/MLKL/p-MLKL. The levels of the necroptosis pathway marker proteins RIP1, RIP3, MLKL and p-MLKL were significantly increased in the HG group compared with the NG group (**Figure 8C** TCMK). After coculture with macrophages in HG, the levels of RIP-1, RIP-3 and MLKL in tubular cells increased significantly (vs. those in the HG group) (**Figure 8C** TCMK). When the cells were cocultured with Notch1-knockdown macrophages in HG, the expression levels of RIP-1, RIP-3 and MLKL were decreased significantly (vs. macrophages cocultured with TCMK-1 under HG) (**Figure 8C** TCMK).

Subsequently, PI/Annexin V stain flow cytometry was used to analyze cell death. The values represent the percentage of cells in each region (PI+ annexin V−: necroptosis, PI+ annexin V+: necroptosis + late apoptosis, PI− annexin V+: apoptosis). The results revealed that the amount of necroptotic renal tubular cells in the HG group was significantly increased compared with that in the NG group. After coculture with macrophages, necroptosis of tubular cells was promoted. After coculture with Notch1 knockdown macrophages, necroptosis was suppressed (**Figure 8D**). Collectively, these results indicate that the Notch pathway plays an important role in macrophages and in mediating renal tubular damage in diabetic nephropathy.

## Discussion

Diabetic kidney disease (DKD), formerly known as diabetic nephropathy (DN), is a major cause of kidney failure worldwide (3, 4). However, the mechanisms by which diabetic nephropathy develops have not been fully elucidated, and the onset and progression of diabetic nephropathy cannot be prevented, despite strict control of blood glucose, blood pressure, and serum lipid levels. With the development of technologies for the pathological analysis of kidney biopsy, the degree of macrophage infiltration in diabetic kidney tissue has drawn attention (7–9). Renal macrophages are closely related to the degree of renal damage, the accumulation of renal interstitial matrix protein and the degree of interstitial fibrosis (7–9). Furthermore, the number of infiltrating macrophages in the kidney and degree of renal macrophage infiltration positively correlate with the level of proteinuria, a decline in renal function in 5 years and disease prognosis (7–9). Diabetic nephropathy is now considered as a chronic inflammatory disease involving macrophages. Our study demonstrated that M1 macrophages in the kidney can secrete proinflammatory mediators such as IL-1β and TNF-α. Additionally, MCP-1, an important mediator of macrophage recruitment (40), increased in mesangial cells and tubular cells cultured under HG conditions. Interestingly, after coculture with macrophages, MCP-1 expression further increased, suggesting the existence of a malignant feedback loop in diabetic nephropathy (**Supplement Figure 8**). Therefore, we propose that under HG conditions, damaged intrinsic kidney cells recruit macrophages into kidney tissue to address tissue damage, but high glucose levels result in macrophage proinflammatory polarization and further produce more inflammatory cytokines. Besides, cell-cell interactions and polarization of macrophages caused further damage to intrinsic cells (**Supplement Figure 8**). Furthermore, we found that macrophage depletion in a classic type 2 diabetic animal model may effectively break this malignant feedback loop. In animal experiments, this effect was initially observed. Macrophage depletion reduced urine microalbumin, blood serum creatinine, blood serum urea nitrogen, and kidney chemokine levels and improved pathological damage to the kidney in diabetic nephropathy mice. The same effect was also confirmed in our *in vitro* experiments.

Macrophages play a pivotal role in kidney injury, inflammation, and fibrosis (41). According to current research, fibrosis is the final result of chronic inflammation (42). Inflammatory macrophages induce fibrosis in response to tissue injury (43, 44). M1 macrophages secrete large amounts of proinflammatory factors, such as IL-1β, IL-6, and IL-10. These cytokines are closely related to inflammation in many tissues. These inflammatory factors are important regulators of the renal inflammatory response and renal interstitial fibrosis (45). Our study also confirmed these findings. After macrophage depletion *in vivo*, the expression levels of inflammatory factors in the kidney tissues of mice were significantly downregulated, pathological changes in the kidney were significantly improved, and the levels of markers of renal fibrosis were significantly reduced. *In vitro*, we found that mesangial cells cocultured with macrophages in a high-glucose environment secreted more of the extracellular matrix proteins FN and Col IV and inflammatory factors IL-1β and IL-18 than that cocultured with macrophages under normal glucose conditions. We considered that in the state of diabetic nephropathy, the excessive and abnormal M1 polarization of macrophages may aggravate fibrosis and loss function of kidney.

Macrophage depletion therapy with clodronate liposomes, as a classic method of depleting macrophages, has been used in the study of various disease models. The current study shows that CL treatment can improve blood glucose homeostasis and insulin sensitivity in obese mice, and attenuate lung injury in rats with severe acute pancreatitis (36, 46). Meanwhile, in the unilateral ureteral obstruction (UUO) mouse model, CL can significantly improve the level of renal fibrosis (47). In our study, we also constructed a long-term continuous macrophage-depleted diabetic nephropathy mouse model. It provides a theoretical reference for the long-term continuous removal of macrophages. While clodronate liposomes deplete all types of macrophages as well as dendritic cells (48). It is an urgent need to find a tissue-specific clearance method to target macrophages in specific tissue.

Notch receptors and their ligands are constitutively expressed in macrophages. The Notch signaling pathway is a highly evolutionarily conserved signaling pathway that was first identified in *Drosophila*. Notch participates in many cellular processes and plays diverse roles. Notch receptors are produced in the endoplasmic reticulum and transported to the cell membrane (49, 50). The interaction between a receptor and its transmembrane ligand causes the proteolytic cleavage of the receptor by the gamma-secretase complex. The cleavage of the receptor results in the release of NICD, which translocate to the nucleus, to exerts its effects. Most evidence has shown that after NICD enters the nucleus, it interacts with the specific transcription factor and DNA-binding protein CSL and then mediates the various effects of downstream factors (51).

However, the latest research has revealed that, in addition to the classic Notch signaling pathway described above, Notch signaling also induces the expression of different genes through crosstalk with other signaling pathways, including the wingless MMTV integration sites (Wnt), transforming growth factor-β (TGF-β), Toll-like receptor (TLR) pathways and signaling pathways induced by hypoxia (52, 53). Specifically, Monsalve et al. found that during NF-κB signaling in lipopolysaccharide (LPS)-treated murine macrophages, Notch-1 expression is upregulated (54). Activated NICD increases the degradation of NF-κB inhibitors and enhances the nuclear translocation and DNA binding of NF-κB, which enhance NF-κB activation in the nornal and LPS-induced macrophage cells. (54). Our study also confirmed the upregulation of Notch-1 expression in macrophages under HG conditions. Luciferase reporter gene, Co-IP and double IF staining experiments have also confirmed that the increase in Notch1 activation further enhances the activity of the NF-κB pathway and its nuclear translocation, leading to an increase in the production of downstream inflammatory mediators.

Recent studies have shown that renal tubular atrophy, tubular cell death and interstitial inflammation promote the development of kidney pathological changes in diabetic nephropathy (55–58), however, the mechanism remains unclear. Some evidence indicates that apoptosis is closely related to a decrease in the number and death of renal tubular cells (59–62), and other evidence indicates that when stimuli are strong, necrosis-like reaction is an important mechanism of renal tubular cell death (63, 64). Interestingly, necroptosis, as a kind of necrosis-like reaction, has emerged as another important mode of cell death during renal tubular damage (29, 65). In this study, we confirmed that renal tubular cells undergo necroptosis in diabetic nephropathy and obtained the same results in experiments *in vitro* by measuring RIP1, RIP3, and MLKL levels, which are the sensitive biomarkers of the necroptosis signaling pathway (65). Furthermore, after coculture with macrophages, the levels of these marker proteins were further increased, indicating that macrophages may participate and promote the necroptosis of renal tubular cells. This phenomenon can be inhibited by the necroptosis inhibitor necrostatin-1.

The necroptosis signaling pathway can be induced by several death ligands, such as TNF, TNF-related apoptosis-inducing ligand (TRAIL), Fas (CD95) and TLRs. TNF has been confirmed to promote the necroptosis of renal tubular cells (65). Based on the evidence, monocytes and macrophages are one of the main sources of TNF and may further promote necroptosis. Therefore, the relation between macrophages and necroptosis have attracted our attention. We found that under HG conditions, the level of TNF-α in macrophages increased significantly. After coculture with intrinsic kidney cells, the level of TNF-α was improved. We further found that this phenomenon is related to kidney cells necroptosis, which is different from apoptosis. It can trigger tissue inflammatory response, thereby aggravating the increase in the secretion of proinflammatory factors by macrophages. These results further prove the hypothesis of the malignant feedback loop we mentioned previously.

In conclusion, in diabetic nephropathy, damaged kidney cells recruit macrophages to alleviate tissue damage. Subsequently, the recruited macrophages are polarized to the proinflammatory M1 phenotype, participate in multiple pathological processes of diabetic nephropathy, including kidney inflammation and fibrosis, and mediate the death of intrinsic kidney cells. The Notch pathway plays an important role in macrophage polarization. When this pathway is activated and can engage in crosstalk with key NF-κB molecules. Furthermore, secretion of downstream inflammatory factors increases, and tissue damage is aggravated (**Supplement Figure 8**). Interestingly, targeting the Notch pathway and macrophage depletion can alleviate tissue damage. Our findings may provide a new direction and new target from the perspective of macrophage and inflammation to treat diabetic nephropathy in the future.

## Data Availability Statement

The original contributions presented in the study are included in the article/**Supplementary Material**. Further inquiries can be directed to the corresponding author.

## Ethics Statement

The studies involving human participants were reviewed and approved by Ethics Committee of the First Affiliated Hospital of China Medical University (approval number: 20202562). The patients/participants provided their written informed consent to participate in this study. The animal study was reviewed and approved by Institutional Animal Care and Use Committee (IACUC) of China Medical University (approval number: 16052M). Written informed consent was obtained from the individual(s) for the publication of any potentially identifiable images or data included in this article.

## Author Contributions

All authors listed have made a substantial, direct, and intellectual contribution to the work, and approved it for publication.

## Funding

The study was supported by the National Natural Science Foundation of China (No. 82070754, 81770724), National Key Research and Development Plan Program-Precision Medicine Research” Special Project (No. 2017YFC0907600), Xing Liao Talents Program Science and Technology Innovation Leading Talent Fund (Distinguished Professor of Liaoning Province) (No. XLYC1902080).

## Conflict of Interest

The authors declare that the research was conducted in the absence of any commercial or financial relationships that could be construed as a potential conflict of interest.

## Publisher’s Note

All claims expressed in this article are solely those of the authors and do not necessarily represent those of their affiliated organizations, or those of the publisher, the editors and the reviewers. Any product that may be evaluated in this article, or claim that may be made by its manufacturer, is not guaranteed or endorsed by the publisher.

## Abbreviations

DKD, diabetic kidney disease; ESRD, end-stage renal disease; NICD, Notch intracellular domain; TNF, tumor necrosis factor; IL, interleukin; iNOS, inducible nitric oxide synthase; WB, Western blot; TUNEL, terminal deoxynucleotidyl transferase-mediated dUTP nick-end labelling; IHC, immunohistochemistry; IF, immunofluorescence; LSCM, laser scanning confocal microscope; HG, high glucose (35mM); NG, Normal glucose (5.5mM).
