# Supplementary material for: Excessive Activation of Notch Signaling in Macrophages Promote Kidney Inflammation, Fibrosis, and Necroptosis
Source: Front Immunol. 2022 Feb 25;13:835879. doi: 10.3389/fimmu.2022.835879 (PMC8913942; doi:10.3389/fimmu.2022.835879)
Supplement: Supplementary file 1 [file DataSheet_1.pdf]

Supplement

Table 1. Primers used for quantitative real-time RT-PCR.

Gene	Primer sequence 5'-3'（Up：Forward，Down：Reverse）
MCP-1	TTTTTGTCACCAAGCTCAAGAG
	TTCTGATCTCATTTGGTTCCGA
CCL8	GAATCAACAATATCCAGTGCCC
	TTGAGACTTCTGGTCAAGGATC
CXCR4	CTCATCCTAGCTTTCTTTGCCT
	GAAGTCACATCCTTGCTTGATG
CXCL12	TCTGAAAATCCTCAACACTCCA
	CAGGTACTCTTGGATCCACTTT
Notch1	GTGCTGGAAGTATTTTAGCGAC
	GTCCTTGCAGTACTGGTCATAC
GAPDH	GGTTGTCTCCTGCGACTTCA
	TGGTCCAGGGTTTCTTACTCC

Table 2. The Antibody used in this Article

Protein	Institution	Catalog Number	Dilution ratio
CD68 (For Human tissue samples)	Abcam Co. (United Kingdom)	ab955	IHC: 1/1500
CD68 (For Mouse samples)	Proteintech Group (China)	28058-1-AP	IHC:1/500<br>WB:1/1000
F4/80	Abcam Co. (United Kingdom)	ab111101	IHC:1/100
iNOS	Abcam Co. (United Kingdom)	ab15323	IHC:1/200<br>IF: 1/200
TNF-α	Novus Biologicals （United States）	NBP1-19532	IHC:1/200<br>WB:1/1000
RIP-1	Proteintech Group (China)	17519-1-AP	IHC:1/200<br>WB:1/250
RIP-3	Proteintech Group (China)	17563-1-AP	IHC:1/100<br>WB:1/500
MLKL	Proteintech Group (China)	66675-1-Ig	IHC:1/20<br>WB:1/200
MLKL (phospho S345)	Abcam Co. (United Kingdom)	ab196436	WB:1/2000
TNF-R1	Proteintech Group (China)	21574-1-AP	WB:1/200
TGF-β1	Proteintech Group (China)	21898-1-AP	IHC: 1:100<br>WB: 1:1000
FN1	Boster Co.	BA1772	IHC: 1:30<br>WB: 1:100
Col IV	Proteintech Group (China)	55131-1-AP	IHC:1/100<br>WB:1/1000
IL-1β	Absin Co.	abs120224	IHC:1/50<br>WB:1/500
IL-18	Absin Co.	abs125418	WB:1/1000

Notch1	Proteintech Group (China)	10062-2-AP	IHC:1/50<br>WB:1/1000<br>IF: 1/20
Notch1	Abcam Co. (United Kingdom)	ab52627	Co-IP:1ug per 200ug total protein
IKK-β	Proteintech Group (China)	15649-1-AP	WB:1/500
NF-κB P65	Proteintech Group (China)	10745-1-AP	WB:1/500<br>IF： 1/20
NF-κB P65	Abcam Co. (United Kingdom)	ab32536	Co-IP:1ug per 200ug total protein
Caspase3	Proteintech Group (China)	19677-1-AP	WB:1/1000
GAPDH	Proteintech Group (China)	10494-1-AP	WB:1/3000
Goat anti-mouse IgG (H+L), HRP conjugate	Proteintech Group (China)	SA00001-1	WB: 1/3000<br>IF:1/2000<br>IHC:1/2000
Goat Anti-Rabbit IgG(H+L), HRP conjugate	Proteintech Group (China)	SA00001-2	WB: 1/3000<br>IF:1/2000<br>IHC:1/2000
HRP-conjugated Mouse Anti-Rabbit IgG, Light Chain Specific	Proteintech Group (China)	SA00001-7L	WB: 1/3000<br>(For Co-IP)
Rabbit control IgG	Byotime Co. (China)	A7016	
FITC-Tyramide Signal Amplification	Servicobio Co. (China)	G1222	IF:1/300<br>IHC:1/300
CY3- Tyramide Signal Amplification	Servicobio Co. (China)	G1223	IF:1/300<br>IHC:1/300

Figure 1. M2 type (CD68+Arg-1+) macrophage cells infiltrated in the kidney of diabetic kidney disease patients is relatively rare.

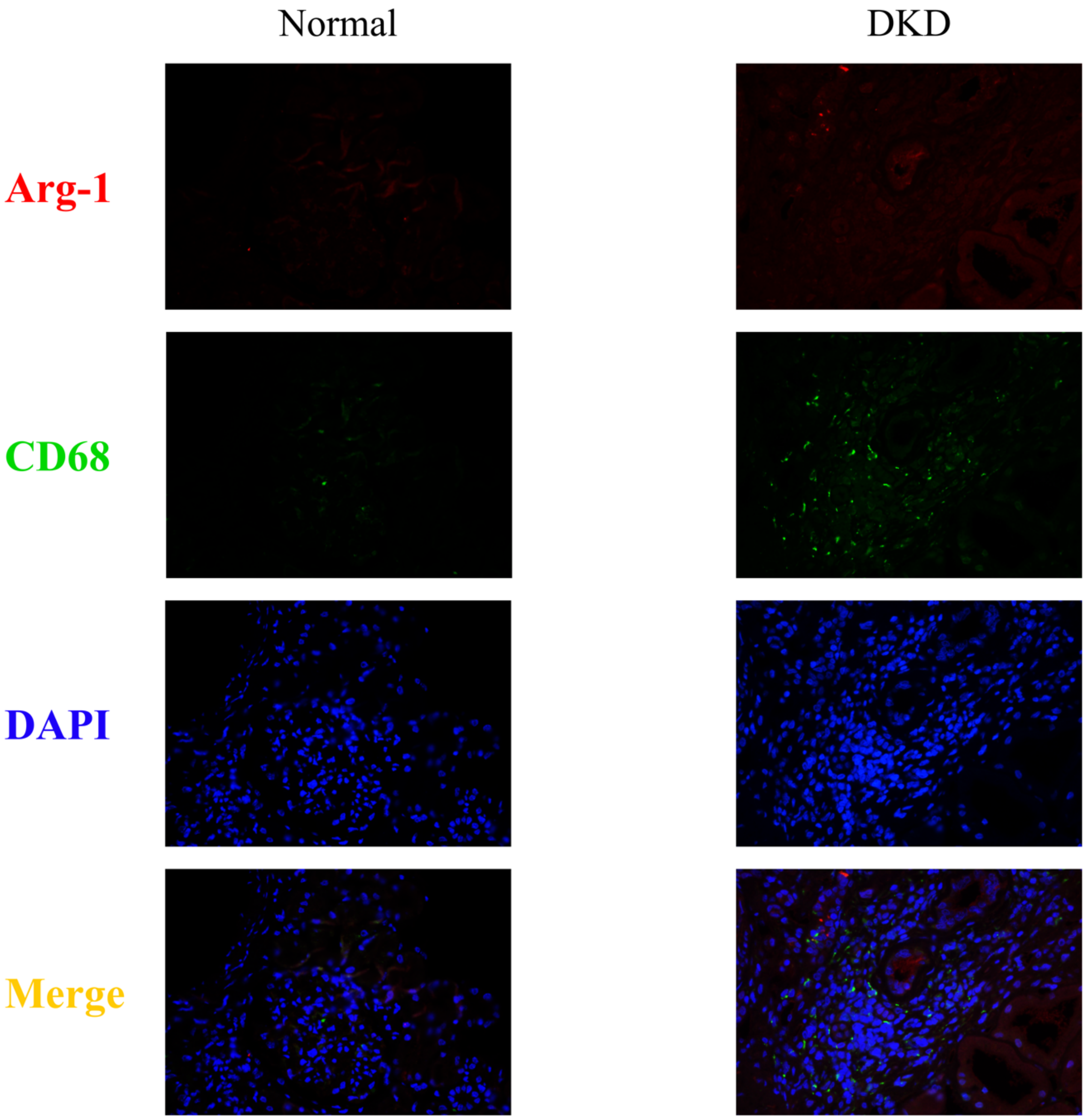

CD68/Arg-1 double immunofluorescence staining. Magnification: ×200. Normal, normal control; DKD, diabetic kidney disease.

Figure 2. Animal experiments have also found that in the mouse model of diabetic nephropathy, the number of M2 macrophages infiltrated is relatively rare.

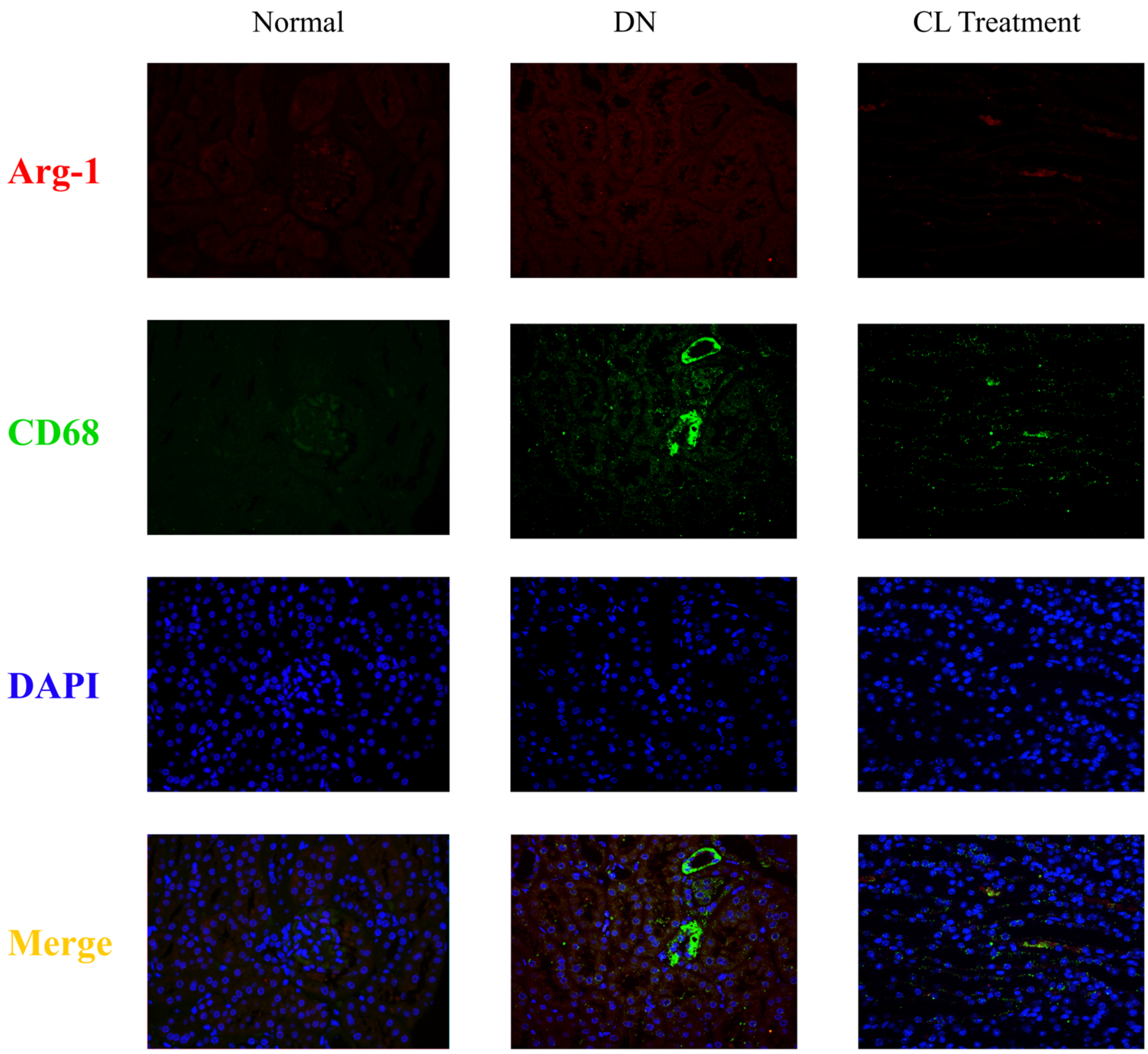

CD68/Arg-1 double immunofluorescence staining. Magnification: ×200. Normal: normal control group (db/m), DN: diabetic nephropathy group (db/db), CL: db/db + clodronate liposome treatment.

Figure 3. Glomerulosclerosis index (GSI) score

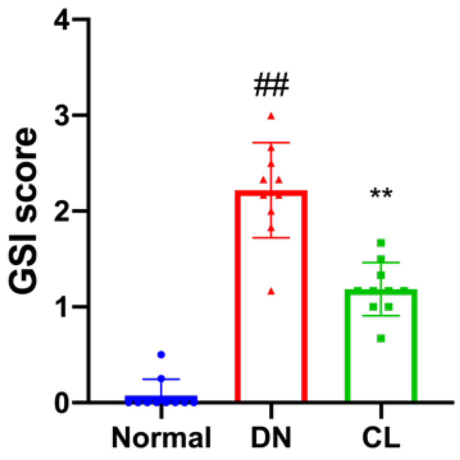

Glomerulosclerosis index (GSI) score of 30-50 glomeruli in diabetic (db/db) / normal (db/m) mouse kidney tissue, mean ± SEM, Normal: normal control group (db/m), DN: diabetic nephropathy group (db/db), CL: db/db + clodronate liposome treatment. # $p<0.05$ vs. the normal group, ## $p<0.01$ vs. the normal group, * $p<0.05$ vs. the DN group, ** $p<0.01$ vs. the DN group.

Figure 4. pNFκB-luc plasmid map：

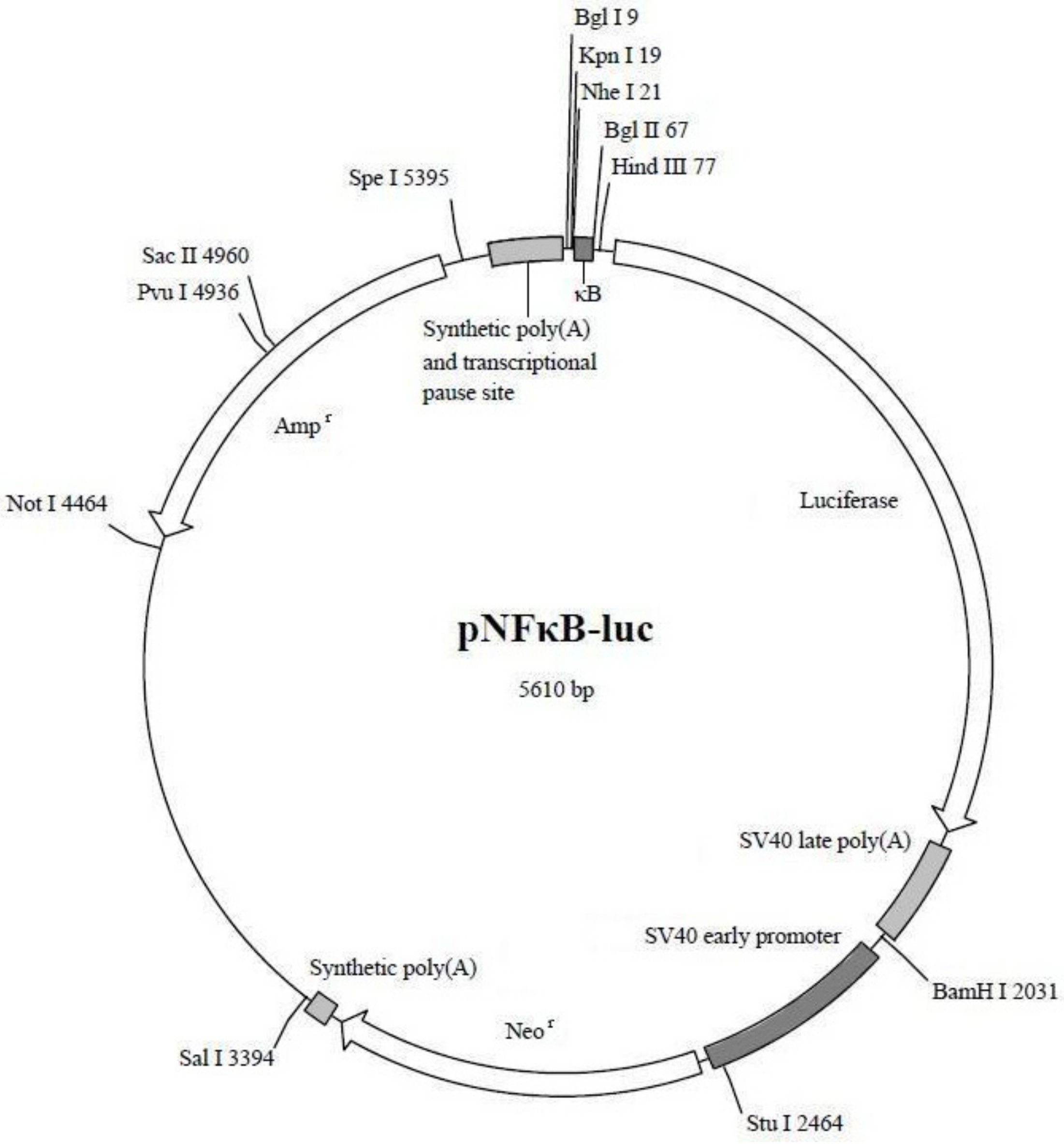

Figure 5. RT-qPCR analysis of siRNA knockdown in RAW264.7 macrophage cell

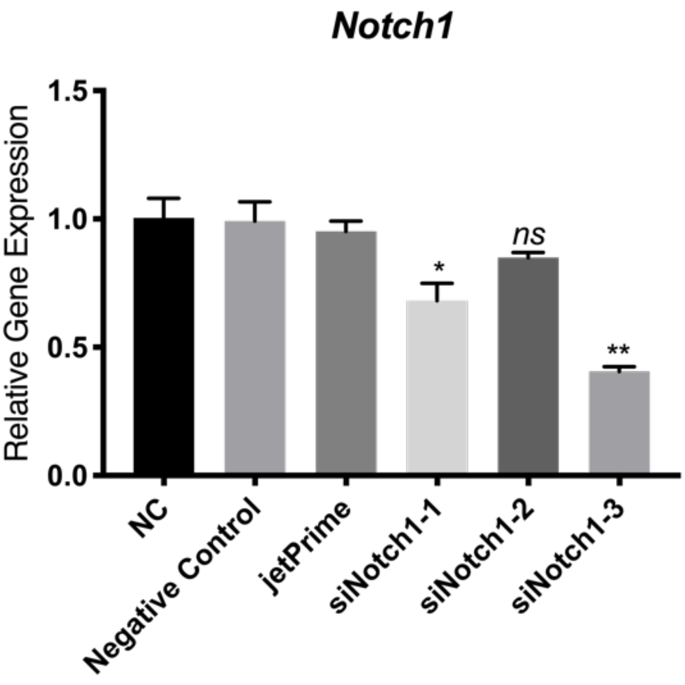

RT-qPCR analysis of siRNA knockdown in RAW264.7 macrophage cell. NC: normal control group, Negative Control: negative siRNA control group, jetPrime: jetPrime control group, siNotch1-1: Notch1 siRNA sequence 1, siNotch1-2: Notch1 siRNA sequence 2, siNotch1-3: Notch1 siRNA sequence 3. ns: no significance vs the NC group, * $p<0.05$ vs the NC group, ** $p<0.01$ vs the NC group.

Figure 6. Notch1 physically interacts with HIF-1α

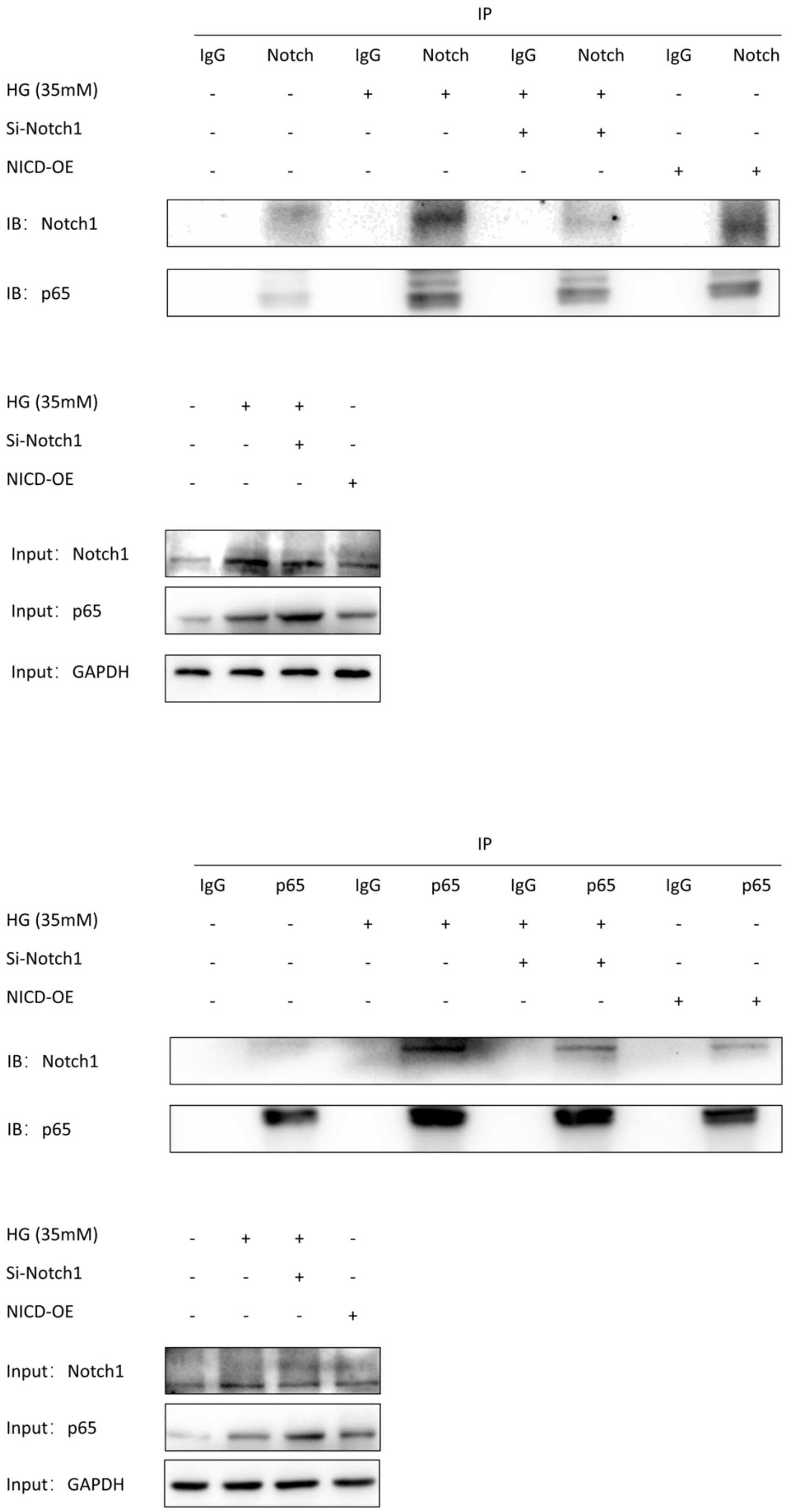

Notch1 physically interacts with HIF-1α. Co-immunoprecipitation (Co-IP) assays of Notch1 and NF-κB p65 in RAW264 cells. Upper part, RAW264 cell lysates were

immunoprecipitated with IgG control antibody, anti-Notch1 monoclonal antibody and then immunoblotted with anti-Notch1 or anti-NF-κB p65 antibody. Lower part, cell lysates were immunoprecipitated with IgG control antibody or anti-NF-κB p65 antibody and then immunoblotted with anti-Notch1 or anti-NF-κB p65 antibody. Notch1 or NF-κB p65 and GAPDH in each group whole cell lysates (input) were shown. (HG: high glucose, NICD-OE: NICD over expression)

Figure 7. HG and necroptosis/apoptosis inhibitors influence the expression of RIP1/RIP3/MLKL pathway of TCMK-1

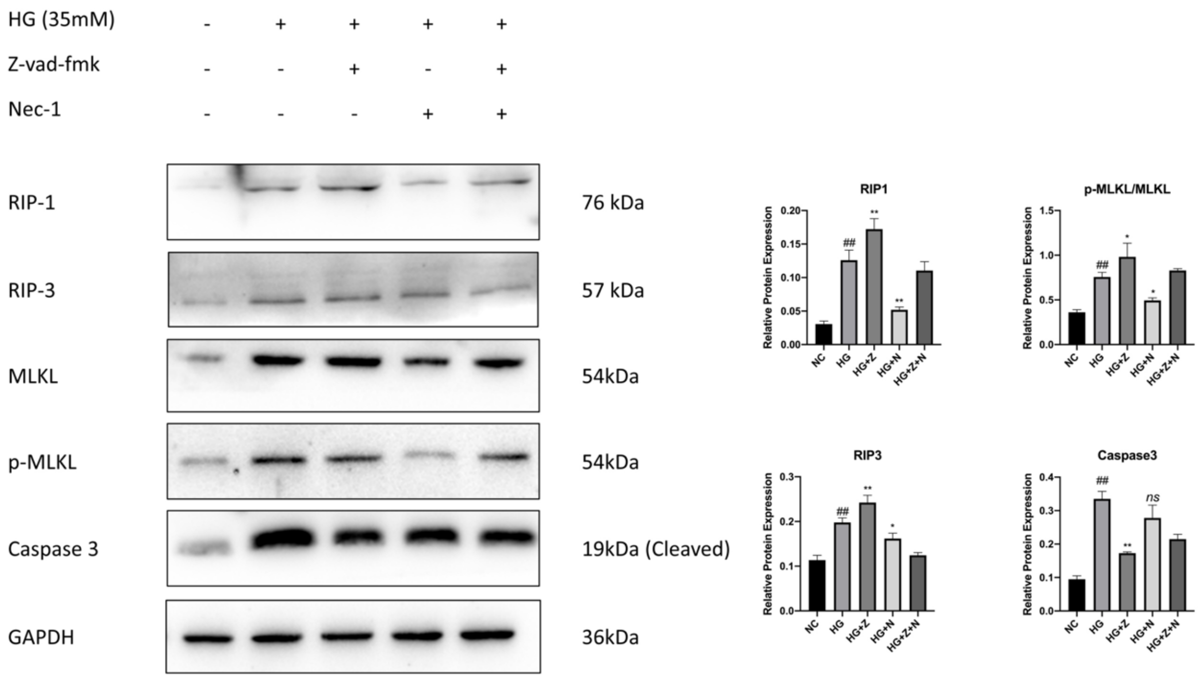

HG and necroptosis/apoptosis inhibitors influence the expression of RIP1/RIP3/MLKL pathway of TCMK-1. Western blots showed that high glucose (35mM) significantly increased the level of RIPK1, RIPK3, MLKL, p-MLKL and cleaved caspase3, Also, the results showed that the expression of RIPK1, RIPK3, MLKL, p-MLKL was significantly higher in TCMK treated with 35 mM glucose+20 μ M z-VAD-fmk than that in other groups, when use of 50 μ M Nec-1 significantly inhibited the expression of RIPK1, RIPK3, MLKL and p-MLKL, but not effect on the expression of cleaved caspase3. (##*P*＜0.01 vs NC, **P* < 0.05 vs HG; ***P* < 0.01 vs HG, *ns* no significance).

Figure 8.

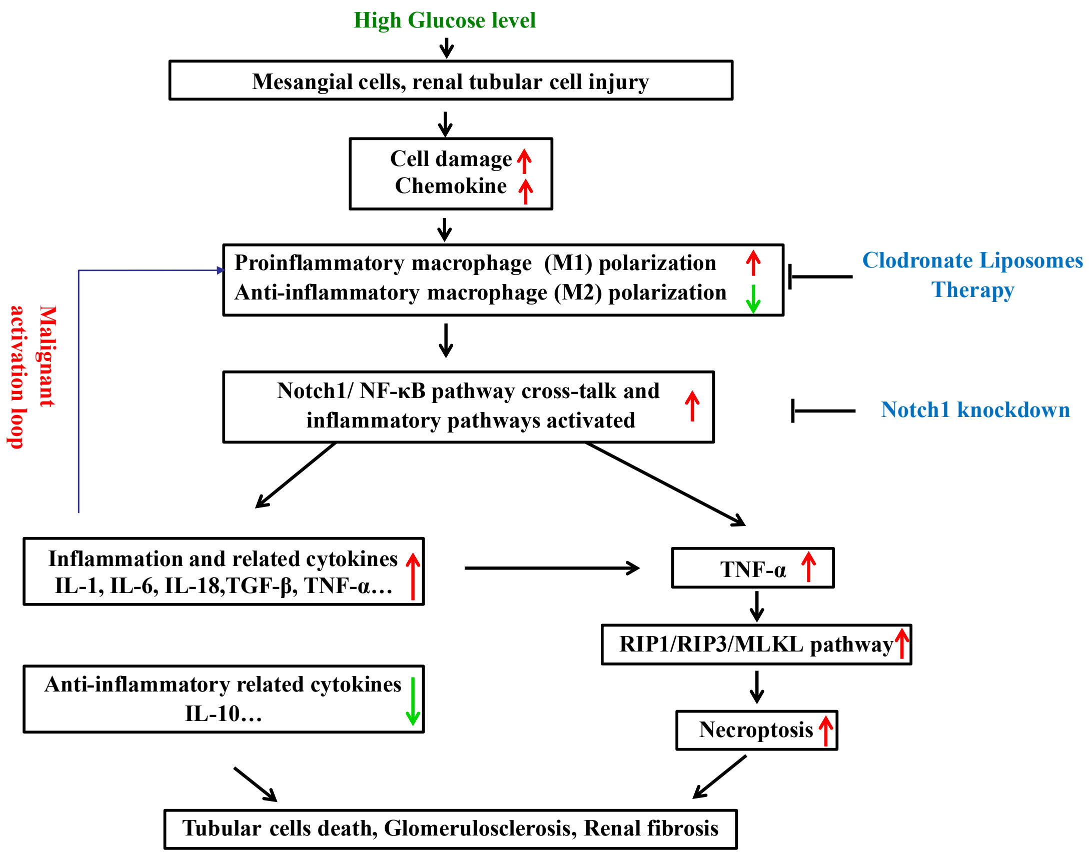

The schematic depicts that high glucose stimulation form malignant feedback loop and leads to excessive activation of Notch signaling in macrophages under diabetic nephropathy. These further mediate kidney inflammation, fibrosis, and necroptosis.
